# When physical activity meets the physical environment: precision health insights from the intersection

**DOI:** 10.1186/s12199-021-00990-w

**Published:** 2021-06-30

**Authors:** Luisa V. Giles, Michael S. Koehle, Brian E. Saelens, Hind Sbihi, Chris Carlsten

**Affiliations:** 1 School of Kinesiology, University of the Fraser Valley, 45190 Caen Ave, Chilliwack, British Columbia V2R 0N3 Canada; 2 School of Kinesiology, University of British Columbia, 210-6081 University Boulevard, Vancouver, British Columbia V6T 1Z1 Canada; 3 Division of Sport & Exercise Medicine, Faculty of Medicine, University of British Columbia, 2553 Wesbrook Mall, Vancouver, British Columbia V6T 1Z3 Canada; 4 Department of Pediatrics, University of Washington, and Seattle Children’s Research Institute, 2001 Eighth Ave, Suite 400, Seattle, Washington 98121 USA; 5 British Columbia Centre for Disease Control, 655 West 12th Ave, Vancouver, British Columbia V5Z 4R4 Canada; 6 Department of Medicine, Faculty of Medicine, University of British Columbia, 2775 Laurel Street, 10th Floor, Vancouver, British Columbia V5Z 1M9 Canada; 7 Legacy for Airway Health, 2775 Laurel Street, 7th Floor, Vancouver, British Columbia V5Z 1M9 Canada; 8 School of Population and Public Health, University of British Columbia, 2206 East Mall, Vancouver, British Columbia V6T 1Z3 Canada

**Keywords:** Walkability, Green space, Air pollution, Heat, Physical activity, Precision health

## Abstract

**Background:**

The physical environment can facilitate or hinder physical activity. A challenge in promoting physical activity is ensuring that the physical environment is supportive and that these supports are appropriately tailored to the individual or group in question. Ideally, aspects of the environment that impact physical activity would be enhanced, but environmental changes take time, and identifying ways to provide more precision to physical activity recommendations might be helpful for specific individuals or groups. Therefore, moving beyond a “one size fits all” to a precision-based approach is critical.

**Main body:**

To this end, we considered 4 critical aspects of the physical environment that influence physical activity (walkability, green space, traffic-related air pollution, and heat) and how these aspects could enhance our ability to precisely guide physical activity. Strategies to increase physical activity could include optimizing design of the built environment or mitigating of some of the environmental impediments to activity through personalized or population-wide interventions.

**Conclusions:**

Although at present non-personalized approaches may be more widespread than those tailored to one person’s physical environment, targeting intrinsic personal elements (e.g., medical conditions, sex, age, socioeconomic status) has interesting potential to enhance the likelihood and ability of individuals to participate in physical activity.

## Background

Precision health, which is in its infancy, involves approaches that both the individual and public health experts can integrate to help protect an individual’s health [1]. It also aims to develop personalized solutions to health problems by considering individual differences in aspects such as environmental determinants of health [2].

Characteristics of built environments and infrastructure, such as transportation systems and greenness, relate to population rates of chronic disease and health inequities [3, 4]. As the physical environment can either facilitate or hinder activity levels [5], physical activity is thought to underlie much of this relationship. Furthermore, less visible features of the physical environment, such as heat and air pollution, affect health independently but also have implications on physical activity [6–9]. Thus, a key challenge in promoting physical activity is maximizing physical activity and its benefits through supportive environments while paying attention to any adverse implications related to a specific physical environment.

It is perhaps intuitive that an understanding of the physical environment-activity nexus will be a powerful tool in precision health if advice and interventions are provided at an individual level; however, research focusing on this is currently in its infancy. Accordingly, we now consider four specific aspects of the physical environment—namely, walkability, green space, traffic-related air pollution, and heat—that could enhance our ability to precisely guide physical activity in the context of the spaces within which we live and move.

## Walkability

### Background

Environments marked by greater density, co-location of residential and retail/services, greater connectivity, and other characteristics including esthetics and better safety are considered more walkable. High walkability environments have residents with higher physical activity [3, 10]. Therefore, those living in more walkable environments attempting to increase physical activity through interventions (e.g., lifestyle counseling, walking groups) may have greater or more sustained increases in physical activity than those living in less walkable environments. Alternatively, those living in less walkable environments attempting to increase physical activity may be able to overcome less favorable environments through intervention or, alternatively, require different interventions to overcome environmental challenges.

### Context motivating precision health

There is limited evidence, with 14 studies identified to date demonstrating no clear pattern of whether the neighborhood environment in which one lives influences physical activity intervention outcomes (Table 1). Some studies found no effect of walkability on physical activity intervention effects [11], while others found greater increases in physical activity among those living in higher walkability neighborhoods [12, 13], and some found greater increases in physical activity through interventions among those living in lower walkability neighborhoods [14].

**Table 1:** Studies examining how participants’ home neighbourhood environment influences physical activity intervention effects

Reference (first author; year)	Setting	Measure(s) of neighbourhood environment		Format of physical activity intervention		Intervention content	Physical activity measurement		Valence of environmental influence on intervention effect		
		Perceived	Objective	Instructor-led	Self-directed		Self-report	Device-based	Augmenting	Overcoming	No effect
King (2006) [12]	5 US metropolitan areas	**√**			**√**	Combined 3 different physical activity trials with varying interventions (e.g., telephone-based counseling, peer group support, culturally tailored self-help)	**√**		**√**		
King (2006) [13]	Primary care facilities in 3 US metropolitan areas	**√**			**√**	3 intervention arms within the Activity Counseling Trial (physician counseling alone, physician counseling plus monthly print materials, physician counseling plus health educator telephone counseling)	**√**		**√**		
Sallis (2007) [68]	Primary care facilities in 3 US metropolitan areas	**√**			**√**	3 intervention arms within the Activity Counseling Trial (see above for details)	**√**		**√**		
Zenk (2009) [69]	Urban and suburban areas in and around Chicago, IL (USA)		**√**		**√**	12-month walking intervention for women in predominantly African-American communities	**√**	**√**	**√**		
Michael (2009) [11]	Portland, OR (USA)		**√**	**√**		6-month lay-led neighbourhood-based walking groups	**√**				**√**
Merom (2009) [70]	New South Wales (Australia)	**√**			**√**	Self-help walking program with weekly diaries with or without pedometers	**√**			**√**	**√**
Kerr (2010) [14]	Urban and suburban areas in San Diego County, CA (USA)	**√**	**√**		**√**	Self-help walking program with weekly diaries with or without pedometers	**√**			**√**	
Gebel (2011) [71]	Wheeling and Parkersburg, WV (USA)	**√**			**√**	Population-wide mass media intervention	**√**		**√**		
Lee (2012) [72]	Houston and Austin, TX (USA)		**√**	**√**		6-month group cohesion intervention to promote walking among African-American and Hispanic/Latino women	**√**	**√**	**√**	**√**	
Barnes (2013) [73]	Perth metropolitan area (Australia)		**√**		**√**	Television mass media campaign promoting 30 min of daily physical activity	**√**				**√**
King (2017) [74]	4 US metropolitan areas		**√**	**√**		Center- and home-based physical activity intervention among older adults	**√**		**√**	**√**	**√**
Jilcott Pitts (2017) [75]	Lenoir County in rural eastern NC (USA)	**√**	**√**		**√**	Four monthly lifestyle counseling sessions, including PA promotion	**√**	**√**		**√**	
Perez (2018) [76]	San Diego County, CA (USA)	**√**		**√**		12 months of *promotoras*-led exercise classes at or near churches and PA promotion provided to Latino women	**√**	**√**	**√**		
Lo (2019 )[77]	Rural towns in MT and NY (USA)	**√**	**√**	**√**		6 months of exercise classes, skills building, and field-based learning provided to women in rural communities		**√**	**√**	**√**	

Note: Instructor-led interventions included some component of structured or organized sessions in which participants engaged in physical activity together led by the intervention team; self-directed interventions included physical activity promotion in various formats (e.g., telephone, web-based, in-person) but did not include structured or organized sessions; see text for description of valence of results

Inconsistency across studies is likely due to differences in environment factors examined and their measurement (e.g., perceived versus objectively derived), differences in physical activity measurement, and differences in sample characteristics. Many existing studies did not originally collect neighborhood environment data and are under-powered to detect potential moderation effects of neighborhood environment. Moreover, these studies were not designed to maximize variability in neighborhood environments in which participants live, although there is a recent exception [15]. There is also limited evidence about whether neighborhood environment influences the maintenance of physical activity gains after intervention ends. Furthermore, these studies assess neighborhood characteristics that might affect individuals, but are less tailored to understanding how the specific characteristics of the individuals (e.g., sex, socioeconomic status (SES)) or changes during intervention (e.g., increased self-efficacy) in those spaces intersects to drive outcomes.

### Conceptual approaches related to precision health

Through better spatial data and geographic information systems, our understanding of and ability to characterize built environment at the individual level has improved substantially and highlights marked differences across individuals in the environments in which they live. Interventions may need to be personalized to the individual’s characteristics (medical, demographic, etc.), the environments in which people spend their time, and the purpose of physical activity that is undertaken (e.g., utilitarian vs. recreational) [16]. For instance, perhaps more self-directed and lower intensity interventions like mass media campaigns could be targeted toward people living in neighborhood environments that are more walkable, as such environments are already supportive of walking. Alternatively, more structured interventions with instructor-led physical activity sessions at safe and accessible facilities may be needed for people living in less favorable environments.

### Current bottom line

Built environment improvements are needed to make physical activity easier and safe, particularly for communities with known inequities in physical activity access and resources. However, given that the built environment changes slowly, a better understanding of how an individual’s built environment influences attempts to increase physical activity, particularly as this may vary based on an individual’s medical, social, and other circumstances, may help to target interventions and improve outcomes. This interest in how walkability influences health behavior changes is growing as we seek to match interventions to the environmental contexts in which people live, work, and play [17].

## Green spaces

### Background

One salient aspect of the physical environment that has received increased scrutiny in the last decade is green space [18]. With the plethora of data on the positive effect of green spaces on various health outcomes [19, 20], a recent theoretical framework offered three general functions to unravel how green space impacts human health [21]: harm reduction (e.g., reducing exposure to heat and air pollution), restoring capacities (e.g., physiological stress recovery), and building capacities, whereby green spaces promote physical activity. Most epidemiological studies demonstrate a positive association between green spaces and physical activity [22], suggesting that green space interventions could be used as a health-promoting strategy, though causality remains uncertain.

### Context motivating precision health

The association between green space and activity may be modified by gender. Compared to men, women seem more likely to use parks and parklands [23], and the likelihood for being physically active is in fact lower for men in neighborhoods with higher green space [24]. This role that gender plays in the association between green space and physical activity may be modified by age as an increase in green space was associated with increases in physical activity in boys but not girls [25].

Given that adolescent health behaviors predict behavior in adults, understanding how green spaces affect physical activity in youth may lead to targeted strategies that can have long-term benefits for adults [26]. Younger school-age children demonstrated higher moderate to vigorous physical activity (MVPA) in school compared with their older counterparts, whereas older boys accumulated more outdoor MVPA in urban green space than younger children [27]. As the age of youth/children affects where they are more likely to exercise, this highlights the importance of targeting specific green space interventions to those who will benefit most (e.g., younger children and school ground greening initiatives).

As highlighted, physical activity can increase with greening [28, 29], and generally, greenness reduces socioeconomic inequalities; however, new evidence suggests that benefits appear to be less in socioeconomically deprived areas [30].

### Conceptual approaches related to precision health

Given that the effects of green space on physical activity can be modified by a number of factors, when planning interventions, it is important to consider the age, sex, and SES of individuals.

As younger children appear to engage in more physical activity at school, targeting nature-based interventions, such as increasing green space in proximity to schools or programs within schools that encourage physical activity in green space, could increase overall physical activity in this age group. In contrast, as adolescents appear to undertake more physical activity outdoors, increasing access and connectivity to green areas around the schools could further increase their overall level of physical activity.

In low socioeconomic areas, the effects of green space on activity appear to be limited; therefore, integrating additional programs to support physical activity is necessary. For example, programs that support safety and address limitations to physical activity such as access to childcare and activity equipment (e.g., bike share programs) would be beneficial. Furthermore, municipal engineers and planners should include additional features (e.g., lighting) that improve a sense of safety and comfort.

Regardless of urban or rural living settings, esthetically pleasing green spaces and physical activity have synergistic effects [31–33]. When investigating the interplay between physical activity and green spaces, aspects such as the restoration pathway, which incorporates stress recovery and increased social contact, should be considered as this pathway may be bolstered by green spaces, in particular for older and younger segments of the population [34–37].

A precision medicine solution could involve a dual approach of combining improvement of the physical environments of green spaces with social engagement elements that are gender and age tailored [33, 38, 39]. However, such a solution would need to consider the geography, climate, cultural preferences, and the makeup of the local population.

### Current bottom line

Increasing green space has the potential to improve health in two key ways. Firstly, green space can modify the physical environment to reduce heat and air pollution and therefore, reduce the physiological burden of heat or air pollution when individuals are physically active. Secondly, green space itself can increase physical activity; however, the potential for this to occur is context specific. Therefore, green space interventions should aim to take into account ways in which different groups (e.g., age, sex, SES) need to be differentially supported (e.g., improvements in safety, access to childcare) or motivated through these interventions (e.g., social engagement).

## Traffic-related air pollution

### Background

The physical environment plays an important role in determining physical activity and air pollution concentrations. In urban environments, densification, street canyons, and high-traffic routes can create air pollution hotspots [40, 41]. In rural environments, woodstove use, backyard, and agriculture burning can also contribute to particular air pollution patterns [42–44].

### Context motivating precision health

As air pollution concentrations increase, individuals, particularly women and those with obesity or respiratory disease, are less likely to participate in physical activity [45–50]. In the absence of data on individual exposure-response relationships, it is unclear if the decrease in physical activity is necessary or wise. Given that the benefits of exercise generally outweigh adverse effects of air pollution [6, 8], many individuals could actually be increasing their health burden by forgoing physical activity.

Facing such uncertainty, individuals are advised to reduce the intensity and amount of physical activity during times of high air pollution, which is typically motivated by “an abundance of caution”. Media alerts and physician advice can play a role in encouraging individuals to modify behavior as air pollution worsens. Specifically, those who received advice from a physician reduced outdoor activity more in response to media alerts than those who did not receive physician advice [46]. Unfortunately, advice to reduce physical activity during poor air quality, in an attempt to minimize short-term harm, may effectively work against the long-term benefits of exercise. Though resolving this balance seems important, there is scarce literature with solid evidence that assesses personalized approaches to exercise in polluted environments.

### Conceptual approaches related to precision health

As exercise is clearly beneficial to physical and mental health [51, 52], it is necessary to understand individual’s goals and risk profile to ensure that, if individuals forgo exercise due to air pollution, these behavior changes are warranted. For example, heavy exercise in areas with high levels of ozone can increase the incidence of asthma in children [53]; therefore, in this subgroup, avoiding exercise during times of high ozone may be necessary. Those with pre-existing respiratory and cardiovascular disease are at an increased risk of the health effects of air pollution, and this risk may be augmented by genetics [54]. Therefore, such susceptible individuals may be counseled to minimize their baseline health risks through lifestyle factors such as diet and physical activity [55].

Since exercising in air pollution can lead to increased discomfort and perceived exertion [56], there is a concern that individuals will be more likely to terminate exercise prematurely. However, it seems that perceived exertion, as opposed to other measures of intensity (e.g., heart rate, running speed etc.), could be a reasonable individual metric to moderate intensities during bouts of air pollution.

Currently, air pollution exposure can be best estimated for large populations through regional modeling, though data is increasingly available for small groups and individuals through personal exposure monitors. Using these monitors in conjunction with smartphones and apps designed to collect health, physiological, and activity (e.g., time-activity patterns) data could facilitate a more precise exercise plan. Specifically, by integrating the use of personalized air pollution monitoring, individuals could receive targeted alerts that are specific to their own health metrics and their own proposed exercise session. However, at the individual level, there are limited data relating air pollution exposure during exercise to health outcomes; therefore, designing such an integrated system will require validation through well-designed research studies. Therefore, planners and officials should consider bringing together scientists with expertise in exposure, health, and exercise science to design and validate tools that integrate personal air pollution exposure with time-activity patterns and individual health metrics.

### Current bottom line

Current knowledge supports more broad-based rather than individually targeted advice on air quality exposure. Specifically, individuals should engage in routine physical activity as this improves baseline health, builds resilience, and therefore, attenuates the risk of air pollution-related health effects [55]. During times of particularly poor air quality, some modifications may be warranted, but exercise need not be forestalled completely—for example, activity in an air-conditioned space will attenuate risk and, if exercising outdoors, separation from traffic is wise.

As women, those with asthma and those with obesity are more likely to attenuate physical activity in areas with high air pollution; particular attention to these groups seems warranted. As evidence grows, personal monitors and apps may prove valid in terms of effectively guiding exercise through integration of pollution data, but caution is warranted; technology- and genetic-driven approaches to personalization in this context are attractive but remain unproven and would need to show clear benefit beyond that associated with non-personalized public health messaging alone. In the interim, guiding exercise through perceived exertion could be a simple personalized approach.

## Heat

### Background

Humans are innately adaptable to activity in hot environments; however, with anthropogenic climate change, mean temperatures and the frequency and severity of heat waves will likely increase.

Healthy humans respond to physical activity in the heat by maximizing heat loss through radiation, convection, and evaporation. When the normal response to heat does not prevent an excessive increase in core temperature, exertional heat illness (EHI) can occur. Heat exhaustion is the most common manifestation of EHI and responds well to rest, hydration, and a cooler environment; however, heat stroke can lead to alterations in level of consciousness, coma, and in rare circumstances even death [57]. Since exercise in the heat can lead to increased discomfort and health risk, there is a concern that physical activity participation levels will decrease [58] if mitigating strategies are not adopted. Therefore, there is a concern that with climate change, physical activity will become riskier and less frequent, especially in vulnerable populations [59], leading to poorer health status for these groups.

### Context motivating precision health

The health risks related to increased temperatures are not evenly distributed, with older adults and children being considered vulnerable populations. With aging, the ability to regulate core temperature is diminished, leading to disproportionately high mortality in those over 50 [60] and especially those over 75 years of age [61]. Young children also have difficulties with thermoregulation in the heat due to their relatively smaller surface area.

Finally, urban dwellers are another population to consider, due to the existence of urban “heat islands,” which occur due to less vegetation, distorted airflow patterns, and anthropogenic heat production leading to increases in daytime temperatures by up to 4° C [62]. Such heat islands inhibit the cooling that typically occurs at night, which can be problematic since night time can otherwise be a convenient time to exercise.

### Conceptual approaches related to precision health

Using personalized physical activity guidance to mitigate the uneven distribution of risks when exercising in the heat is a challenge since so many factors play a role in heat tolerance. Genetics may increase the risk for EHI; therefore, there is the potential for genetic screening to identify personalized heat illness risk, and therefore, in these groups, special attention could be paid to physical activity guidance in the heat [63]. Core temperature is the most important variable to measure, but this is not straightforward. The most proven and usable sensors, pills that transmit temperature to an external reader, are used primarily in research and are not practical for use by consumers. Many wearable devices (such as smart watches and bracelets) have temperature sensors, but these do not accurately capture core temperature. Wearable sweat sensors, with the potential to measure not only electrolyte content but also other sweat constituents, may play a role in mitigating risk by guiding hydration and nutritional intake. However, it is unclear whether this is superior to using urine color

Heat acclimation is critical to safety at elevated temperatures. With exposure to heat, an adaptive response lowers core temperature. Since core temperature generally rises during physical activity, exercise training even under temperate conditions provides significant acclimation. Thus, minimizing sedentary behavior year round and promoting physical fitness are critical. Secondary recommendations include wearing appropriate clothing during activity that maximizes heat loss and ensuring adequate hydration during and following physical activity [64].

All of the above are important especially for at-risk community members, though each individual’s limits need be respected. For example, in those with impaired kidney function, dehydration may pose particular risk, while overhydration can be a challenge for those with congestive heart failure.

Public awareness programs around safe practices in the heat with regards to physical activity should be a key role of governments in this area. The response to previous heat waves is illustrative. Following the 2003 heat waves in France (14,800 deaths in 3 weeks [65]), the French government instituted a nationwide alert system that reduced mortality in subsequent events [66]. A telephone hotline, targeting the vulnerable, offered emergency assistance and provided advice, including limiting physical activity [67]. From a planning point of view, mitigating the urban heat island effect by maximizing the presence of vegetation and employing lighter colored materials on exposed surfaces should be a primary consideration.

### Current bottom line

Heat is a stressor that is generally well tolerated but can pose particular threats to health with physical activity. Advice regarding prevention of heat-related illness (acclimatization and hydration) is broadly applicable, but personalized approaches include providing extra caution to at risk groups such as the elderly, children, and those with kidney and heart disease. Additionally, consideration of personal temperature-, electrolyte-, and heart rate-based prescriptions (subject to pending evidence of effectiveness), and individualized advice responsive to circumstance such as local climate, urban geography, and temporal flexibility will be beneficial.

## Conclusion

In order to derive the considerable health benefits from physical activity, populations need to be more physically active. It is well documented that there is variability in response to physical activity and exercise interventions. To advance both the science and application of physical activity and exercise, moving beyond the “one size fits all” to a precision-based approach is critical. Personal characteristics such as self-efficacy, health metrics, sex, age, SES, and genetic predisposition may make an individual more or less likely to succeed in attempts to increase physical activity. However, as suggested by ecological models, the physical environment may also influence such attempts, and so we have considered the role of physical environment in the process of evolving to that more individualized “prescription”.

It is worth emphasizing a few key points that emerge from the four themes we have explored. First, in some cases, generic (non-personalized) approaches may be more effective, or at least more cost-effective, than those tailored to one person’s physical environment. It is possible that trying to use environmental context to give precise individualized activity will in some settings prove unnecessarily complex or confusing. Moving forward, we will need to seek evidence carefully and specifically to prove that precision works.

That said, we are optimistic that the benefits of precision health for physical activity in the context of the physical environment will become clear. Ultimately, the accessibility, features, condition, and actual and perceived safety of their surrounding environment influence engaging in any type of physical activity. However, to orient efforts moving ahead, it is useful to take into account personal elements (e.g., medical conditions, sex, age, SES, self-efficacy) and how these interact with the physical environment to modify the benefits of or likelihood to participate in physical activity.

In some situations, semi-personalized advice (e.g., to groups with particular conditions, barriers, or environmental constraints in common), rather than that precisely individualized, may be more pragmatic

Finally, we recognize that there are many factors related to the physical environment that we have not considered here; however, these four factors were chosen to provide a breadth and depth of information that will be useful for others moving forwards. In future, more research will be needed to determine the effectiveness and feasibility of providing greater precision to exercise recommendations in relation to the physical environment.

## Data Availability

Data sharing is not applicable to this article as no datasets were generated or analyzed during the current study.

## Abbreviations

SES: Socioeconomic status
MVPA: Moderate to vigorous physical activity
EHI: Exertional heat illness
